# Perceiving politicians as true to themselves: Development and validation of the perceived political authenticity scale

**DOI:** 10.1371/journal.pone.0285344

**Published:** 2023-05-24

**Authors:** Simon M. Luebke, Ines Engelmann

**Affiliations:** Institute of Communication Science, Friedrich Schiller University, Jena, Germany; St John’s University, UNITED STATES

## Abstract

The authenticity of political candidates receives increasing attention in political and academic discourse. Although being perceived as authentic is seen as a success factor in contemporary political communication, little attention has been paid to how citizens evaluate politicians’ authenticity. The state of research thus lacks a valid instrument to measure citizens’ perceptions of politicians’ authenticity. This article addresses this gap in the literature and proposes a new multidimensional scale of perceived political authenticity. We conducted three consecutive studies to test the instrument’s composition, performance, and validity and present a final 12-item scale. Results from an expert panel and two online quota surveys (Sample 1: *N* = 556, Sample 2: *N* = 1,210) show that citizens rely on three political authenticity dimensions to judge politicians’ authenticity: ordinariness, consistency, and immediacy. Factor analyses were used to establish construct validity and demonstrate that the new scale is a robust and reliable measure. Finally, we find that higher perceived political authenticity for specific politicians is positively associated with party identification and the intention to vote for politicians.

## Introduction

The popular image of a good politician held by citizens has changed. This statement captures observations by Clarke et al. [1] that the notion of good politicians and the criteria to evaluate political candidates among citizens today differ from those of the mid-twentieth century. While citizens were said to desire politicians who are honest, strong, and hard-working, they now also expect candidates to be normal, ordinary, and “in touch with reality” ([1], p. 207). The authors summarize this desire for truthful politicians under the term of authenticity, arguing that citizens increasingly want politicians to be (more) authentic [1,2]. Their conception of authenticity is rather narrow and leaves out many of the concept’s facets, such as genuineness, spontaneity, and showing one’s true self. Yet, their observations indicate a growing relevance of political authenticity among citizens [3,4]. Although it is far from being a new concept in politics [5], authenticity plays an increasingly decisive role in political campaigning and elections [6–10]. Kenny et al. [11], for example, note that scholars and journalists frequently refer to politicians’ authenticity to explain why voters prefer certain candidates over others. Brewer et al. [12] consider authenticity as a key trait in citizens’ candidate judgments, whereas Seifert ([5], p. 209) even discusses the rise of “a new age of authenticity” in politics. Furthermore, Ceccobelli and Di Gregorio [13] see authenticity as a dimension of a triangle of leadership that explains the popularity and success of contemporary political leaders. Thus, being not perceived as fake but as authentic is an important aspect in voters’ evaluations of political candidates [2] and considered “critical to political success” ([14], p. 318). Recent studies support this assumption and suggest a positive relationship between perceived authenticity and the intention to vote for candidates or their parties [15]. Accordingly, politicians across the political spectrum try to perform the role of authentic candidates [16]. This seems to be especially successful among politicians from populist parties, who are said to have an aura of authenticity due to their roles as political outsiders [7].

Despite the concept’s relevance in political communication, authors recognized that only little research investigates audience perceptions of politicians’ authenticity [5,17]. We assume that this can be explained by the term’s “indeterminate nature” ([18], p. 90) and the lack of valid instruments to measure and compare perceived political authenticity. Empirical research has neither agreed on a common definition for perceived political authenticity nor on dimensions and indicators to measure the concept. So far, poll and survey studies relying on different instruments outlined *that* citizens perceive some candidates as more authentic than others [7,19]. However, even if people seem to differ in how they perceive politicians as true to themselves, we hardly know *how* these differences between recipients are constructed. For example, do people who perceive one politician as authentic (e.g., Donald Trump) apply the same criteria to judge the authenticity of his or her opponent (e.g., Joe Biden)?

This article addresses this methodological gap in the literature and proposes a new scale for measuring audience perceptions of politicians’ authenticity. Hence, we build on the conceptualization of political authenticity offered by Luebke [4] to measure perceived political authenticity as a multidimensional social construct. We define perceived political authenticity as the degree to which politicians are perceived as true to themselves. Hence, political authenticity is not a quality that politicians possess, but an impression of observers that they are true to their inner selves [20]. Following this definition, we conducted three consecutive studies to develop a German scale and test its composition, performance, and validity in two online quota surveys in Germany. Furthermore, to understand the dynamics around perceived authenticity in politics, we also examine its relationship with relevant antecedents (e.g., party identification) and outcome variables (e.g., vote intention) previously mentioned in the literature.

### Perceived political authenticity

Authenticity is considered an important candidate trait of politicians that receives increasing attention in political communication research. However, research on political authenticity lacks a common definition of the concept, which is often mistaken with integrity or sincerity. Thus, researchers associate different aspects with authenticity. Some scholars define political authenticity as the congruence between politicians’ shared positions and their actual views [3,18,21]. In the Goffmanian sense, they understand political authenticity as “a minimal difference between the frontstage persona presented to the public and the backstage persona presented to intimates”([21], p. 29). Other authors associate political authenticity with politicians’ “realness” [7,15,16]. Political candidates are considered authentic when their positions are genuine and do not aim to manipulate voters [16]. Finally, scholars refer to political authenticity as politicians’ approachability and ordinariness [2,22]. Authentic candidates are said to appear as down-to-earth people who are part of the community "rather than being detached and part of an elite" ([23], p.39).

Despite their differences, the various conceptions of political authenticity in political communication research share the view that the concept deals with the degree to which politicians are and remain true to themselves. This is also the conclusion of Luebke’s [4] synopsis, which reviewed previous literature on authenticity in politics. In line with his notion of political authenticity, we define perceived political authenticity as a subjective assessment by citizens whether politicians appear as true to themselves. Whether politicians appear as true to their inner selves can be based on different impressions. Accordingly, we assume that politicians appear true to themselves when their actions are consistent with their true views (consistency), when they reveal intimate insights about their lives (intimacy), appear down-to-earth (ordinariness), and seem not to be influenced by others but do and say what they think (immediacy) [4]. This conceptualization allows distinguishing political authenticity from concepts such as integrity and sincerity, which have often been confused with authenticity in prior work [18]. Integrity describes the idea that politicians act according to morally right principles, whereas authenticity means they act in line with their own principles whether or not they are morally right [3,15]. Likewise, while being sincere means an accurate representation of beliefs to others, the authenticity concept is narrower and describes the accurate representation of the beliefs that define politicians’ own selves [3,4]. Authenticity thus describes a concept that is not necessarily about acting true to the facts, but about being true to oneself. Still, both concepts have been part of definitions and operationalizations of perceived political authenticity in prior research, making the often vague authenticity concept even more elusive.

Perceptions of politicians’ authenticity are unstable and contextual interpretations by the audience [24] based on information from the media, such as politicians’ performed political authenticity (e.g., their self-presentation in social media) and the media’s construction of authenticity (e.g., candidate portrayal by newspapers) [4,25]. Citizens also rely on existing political attitudes and experiences to judge candidate authenticity [9]. As people will not know for sure whether politicians act in line with their true selves [26], authenticity judgments are subjective probability statements [18].

Research has shown that some citizens perceive the same performances by politicians as more authentic than others [19]. These differences in perceived political authenticity result from perceivers’ ideology, political attitudes, and media use [9,12,27,28]. Consequently, citizens perceive a specific politician to be more authentic when they identify with a candidate’s party. Perceived political authenticity was also shown to be associated with higher exposure to different political information, such as TV news and candidate profiles in social media [12,29,30].

### Dimensions of perceived political authenticity

In line with the definition presented above, the literature proposes four dimensions of political authenticity, which serve as starting points for our operationalizing of the concept: consistency, intimacy, ordinariness, and immediacy [4]. We assume that each dimension captures similarly relevant impressions to judge politicians’ authenticity.

*Consistency* describes perceptions of politicians as true to themselves by acting consistently and according to expectations. It assumes that politicians appear authentic when they perform similar actions over time and space, e.g., by sticking to their opinions, statements, and narratives [31]. Politicians are perceived as true to themselves when their actions correspond with the individual expectations recipients have of them [5]. Recipients have expectations based on former experiences with this politician, politicians of his/her kind (e.g., same party/ideology), or politicians in general [4].

The *intimacy* dimension assumes that citizens perceive politicians as more authentic when they believe to know politicians’ true characters and real personalities behind their public figures [16]. This dimension reflects various perceptions of politicians’ private persona [6]. Assuming that politicians are more likely to show their real selves when less exposed to public expectations, their private persona is often associated with their authentic selves. Authentic politicians care about openness to others by sharing private thoughts and views [20,29], whereas candidates who keep their private lives out of the public eye are more likely to appear less authentic.

The *ordinariness* dimension assumes that politicians are considered authentic when they appear as ordinary people like you and me [4]. The perceived ordinariness contradicts the image of the inauthentic and professional statesman or -woman. Politicians are perceived as authentic when they appear not aloof but close to the people from our communities and daily lives [32]. Accordingly, authentic politicians seem like down-to-earth people who do not change even if they are in power. When politicians have minor flaws and show moments of imperfection, they appear more authentic [20].

Finally, the fourth dimension of political authenticity is *immediacy*. Perceived immediacy results from impressions that politicians directly reveal their true selves to the public [8]. This applies when the actions of politicians do not seem strategic but rather driven by their true convictions and emotions. Politicians are perceived as immediate when they appear spontaneous and say what they think, regardless of the consequences. Politically incorrect speech is thus often considered as indicator of immediacy. When politicians do not mince matters, they appear less controlled or strategic and thus more authentic [27].

### Measures of perceived political authenticity

Researchers have not agreed on a standard measure of perceived political authenticity but relied on different single items, one-dimensional, or multidimensional measures. Hahl et al. [19], for example, applied a *single-item measure* and asked respondents to rate politicians’ authenticity on a scale of 1 (low) to 7 (high). Brewer et al. [12] used the words “authentic” and “phony” to assess the authenticity of US presidential candidates. Enli and Rosenberg [7] asked respondents to rank the “realness” of Norwegian politicians and interpreted the highest-ranked politician as the most authentic candidate.

More studies assume a *one-dimensional* authenticity construct and investigate authenticity with multi-item scales. This includes measures that combine the single item “authentic” with items of similar constructs, such as “sincere”, “genuine”, or “honest” [14,33]. Stiers et al. [15] suggest six authenticity items (transparent, bold, consistent, conviction, honest, truthful), which loaded on one authenticity factor. Other one-dimensional measures rely on items from the Authenticity Scale (AS) [34], the Authenticity Inventory (AI) [35], and the Authentic Leadership Questionnaire (ALQ) [36]. As these scales were developed to measure authenticity as a self-experience (“Am I authentic?”), scholars adjusted these scale items to investigate interpersonal authenticity [27,28].

Finally, studies use scales for perceived authenticity that are either assumed or found to be *multidimensional*. These scales rely on items from either psychological or political science research. The General Authenticity Scale (GA) by Pillow et al. [9] is part of the first strand. It combines AS and ALQ items with single-item ratings (authentic, genuine, trustworthy, transparent, honest). The second strand of research is inspired by Louden and McCauliff [18], who propose twelve potential questions for authenticity judgments derived from three dimensions: personal qualities and traits, competence, and communicative behavior. The items were not empirically tested by the authors but have been adopted and applied by scholars to measure perceived political authenticity [37,38]. The application of the scale shows that items from all three dimensions load on only one latent factor [38].

We believe that these measures have various *limitations*. First, we argue that single authenticity items do not cope with the proposed multidimensionality of political authenticity. Using a single authenticity item ignores the complexity of the social perception processes suggested by the literature. Assuming that respondents have different associations with the term “authentic”, single items do not allow examining or comparing the ideas and understandings citizens base their judgments on. Some authors thus applied multi-item one-dimensional measures, which combine single authenticity items with items for alleged synonyms, such as perceived sincerity or genuineness [14,15]. However, these approaches neither acknowledge the concept’s multidimensionality nor its conceptual distinctions from related concepts like integrity, sincerity, or honesty assumed in the literature [3,15]. We further argue that the few multidimensional scales in the literature lack a clear theoretical basis and validity for measuring perceived political authenticity. Although these scales possess high internal consistencies, their actual dimensionalities and compositions have rarely been examined. This is especially inconvenient when scale items were originally developed to measure authenticity as a self-experience. Whether these items are also adequate and valid to measure intersubjective perceptions of politicians’ authenticity must be examined. For the above reasons, we conclude that research on political authenticity would benefit from a new scale, which reflects the construct’s multidimensionality and has been validated to measure audience perceptions.

### Methodological approach for scale development and validation

The Perceived Political Authenticity (P-PA) Scale was developed in three consecutive studies, which were conducted in line with scale development guidelines [39–41]. The first study served the purposes of item generation and content validity assessment. The second study used a quota survey to identify the adequate number of factors and establish preliminary construct validity (*N* = 556). Finally, the third study used data from a second independent quota survey (*N* = 1,210) to confirm the scale’s factor structure and examine its relationship with relevant antecedents and outcome variables in a nomological network.

### Study I: Item generation and content validity

An initial pool of scale items was developed to represent each of the four theoretical construct dimensions effectively. As we operationalized the construct using dimensions proposed in the literature, the item generation process in this study followed a deductive approach [40].

### Item generation

The items were either adopted from existing scales or developed specifically for the study. Items from existing scales were included when they dealt with the authenticity of persons and represented one of the four dimensions of political authenticity. The items were also checked to ensure that they were worded simply and not ambiguously [39]. However, no existing item was excluded due to its wording. These criteria applied to 16 items from existing measures for perceived authenticity of politicians [15,27,38], celebrities [28,42], and people in reality TV programs [43]. Most items were adopted without changes. As these 16 items did not reflect all relevant facets of each dimension outlined in theory, we developed 24 new items, complementing an initial pool of 40 items (S1 Table). At least one item in each dimension was designed as opposed to its designated construct to avoid an agreement bias. Because the validation studies were conducted in Germany, two bilingual translators, one concept expert and one naïve translator, who was unfamiliar with the concept, translated the items into German and agreed on a joint version.

### Content validity

The content validity of all items was assessed to ensure that the initial item pool adequately reflects the desired constructs [40]. We used a more elaborate content validity approach, which captures both qualitative and quantitative feedback of content and method experts [41]. Six expert judges completed an online questionnaire in which they gave qualitative feedback on items’ clarity, necessity, and comprehensiveness (see S2 Table for details on the expert panel and the questionnaire). We also asked the experts whether they thought each item corresponded to the four dimensions of political authenticity using a four-point ordinal rating scale (1 = not relevant, 2 = somewhat relevant, 3 = quite relevant, 4 = highly relevant) [44].

The experts were first given an overall definition of political authenticity. Second, they read a definition of authenticity as either consistency, intimacy, ordinariness, or immediacy and evaluated whether each item matched the presented definition. This was repeated four times, each time with the definition of a different authenticity dimension [45]. The order of definitions and the order of items at each page were rotated to avoid order effects. The experts were allowed to assign items to more than one dimension. Items’ assignment to several dimensions either indicated item ambiguity or a lack of dimensions’ discrimination.

The item-level content validity index (I-CVI) was calculated to quantify the experts’ ratings and to report the proportion of experts who assigned an item to the designated dimension [45]. Experts’ proportional agreement was calculated by first dichotomizing their ratings into content invalid (1 and 2) and content valid ratings (3 and 4), and then dividing the number of valid ratings by the total number of experts [46]. Due to the complexity of the authenticity construct and the size of the expert panel, we did not expect a perfect agreement between all experts [47]. Items on which four out of six experts agreed (I-CVI values ≥ .67) were considered to have fair content validity and were only slightly reworded depending on the qualitative feedback [47]. Items with I-CVI values lower than .67 were either discarded or revised based on the experts’ qualitative comments. I-CVI values for the items ranged from .17 to 1 and resulted in the deletion of six items and the revision of nine items (S3 Table).

All nine substantially modified items were tested again in a second expert panel of three senior communication scientists (two female and one male) from two universities in Germany who weren’t experts in the first panel [47]. Second round I-CVI values ranged from .33 to 1. Again, items with values ≥ .67 were kept, whereas one item that ranked below the threshold was deleted. The final list consisted of 33 content valid items (S3 Table). Nine of these items were assigned each to the consistency and ordinariness dimension, eight to intimacy, and seven items to immediacy. The average CVI for all subscales (S-CVI > .84) and the total CVI for the overall scale (S-CVI = .89) indicated good content validity [47]. The remaining items were back translated to English by two bilingual translators and double-checked by an experienced English speaker.

### Study II: Exploratory factor analysis

#### Sample and procedures

All content valid items were included in an online quota survey of the German adult population. The participants were recruited via an online access panel by the professional survey company *respondi AG*. Respondents participating in the survey received a financial incentive. They were informed about the study’s purpose and procedure, their right to decline participation and the confidentiality of their responses. Participants gave informed consent by accessing the online questionnaire. The written consent was recorded by the survey software. To ensure that the sample reflects the German adult population above 18 in its basic demographics, we used a quota procedure for age, gender, and education (S5 Table). The full questionnaire was pretested with a convenience sample (*N* = 30) and had two instructed response items (IRI) as attention checks to ensure data quality. Participants that fit the quota requirements were excluded when they failed an attention check question, needed less than half of the sample median time to complete the survey, or implausibly straight-lined the authenticity items. After the exclusion of inattentive participants, speeders, and straight-liners, 556 cases (age: *M* = 46.5, *SD* = 15.9, range = 18–74; 50.4% female) remained for the analyses (for details see S4 Table).

As political ideology impacts authenticity perceptions [12], authenticity ratings based solely on preferred candidates may have been biased and have low variance. Thus, we provided respondents with a list of twelve male and female politicians who were among the best-known German federal politicians at the time of the study and members of one of the six German parties in the parliament (S6 Table). The order of politicians’ names was rotated. We further assumed that citizens must have formed impressions of politicians from available information to make valid statements about their authenticity. As most citizens do not know politicians personally [7], we first asked respondents how well they knew various German federal politicians from the media (S7 Table). Of the politicians whom each respondent knew at least well (≥ 3), one was randomly selected, and respondents completed the 33 randomly rotated P-PA items regarding this candidate. All P-PA items were measured on a five-point Likert-type scale, which was labeled at the two endpoints and ranged from 1 (I completely disagree) to 5 (I completely agree).

#### Data analysis

First, we examined the univariate normality, difficulty, and discrimination of all P-PA items. The analysis of univariate normality shows that several items were moderately skewed (< 1) or had moderate negative kurtosis (< 1). Mardia’s coefficients for multivariate skewness and kurtosis suggested deviations from multivariate normality (skewness: *z* = 15,826.85, *p* < 0.01; kurtosis: *z* = 82.05, *p* < 0.01). All items showed a satisfactory level of item difficulty (*index p* > .20 and < .80). Five items, including four reverse coded items, had negative or very low item discrimination values (r_itc_ < .1) and were excluded from the analysis (con_4r, int_7r, ord_8r, ord_9, imm_10r). The poor performance of the reverse coded items suggests that these items opposed to the construct may have measured a different construct than authenticity [48]. Due to violations of the normality assumption, we applied an exploratory principal axis factor analysis (PAF) as a robust method to examine the underlying factor structure [41]. Although the item development was based on the hypothesized structure of an a priori model, it is suggested to perform EFA for an instrument’s initial validation [49]. The PAF was calculated using oblique rotation (Promax) because we expected the scale dimensions to be correlated. Parallel analysis (PA) was conducted to determine the adequate number of factors. PA was shown to be more accurate in estimating the number of factors than the eigenvalues-greater-than-one rule or scree tests [50]. PA and PAF were both calculated using R package *psych* [51].

#### Results

We first tested the factorability of the 28 items. The Kaiser-Meyer-Olkin (KMO) test for sampling adequacy (overall MSA = .96; MSA for each item > .56) and Bartlett’s test of sphericity (*χ*^*2*^ (378) = 10165.8, *p* < .01) indicated applicability of the factor analysis. The PA with 28 items suggested a four-factor solution (S1 Fig). The PAF exhibits a good fit for a four-factor model (χ^2^ (272) = 655.39, *p* < .001, CFI = .962, RMSEA = .050 [90% CI = .044 - .056], SRMR = .028). However, one item had no salient loadings for any of the factors (int_5, λ < .40), whereas a second item had salient cross-loadings (imm_2). After the exclusion of both items, another PAF with 26 items was calculated. It exhibits again a good model fit (χ^2^ (227) = 548.65, *p* < .001, CFI = .963, RMSEA = .050 [90% CI = .044 - .056], SRMR = .028) and showed no cross-loadings.

The four factors explained 57.7 percent of the items’ variance. The first three factors comprise at least five items and had high Eigenvalues (> 2.7), whereas the fourth factor comprises only two items and hardly contributes to the explained variance (Eigenvalue = 1.0). Two items addressing politicians’ imperfection as one facet of ordinariness show the strongest loadings for this factor. Although imperfection was considered an aspect of ordinariness, both items show only weak correlations with the other ordinariness items (*r* < .15). The weak latent correlations between this fourth factor and the other three factors (*r*_*1*_ = .07; *r*_*2*_ = .26, *r*_*3*_ = .02) suggest only little shared variance. Thus, we decided to remove the two items (ord_6, ord_7) and run a third PAF with the remaining 24 items. Factorability of the items was again strongly given (overall MSA = .96; MSA for each item > .88; Bartlett’s test: *χ*^*2*^ (276) = 8863.87, *p* < .01). The PA now suggests three factors (S2 Fig). PAF also exhibits a good fit for a three-factor model (χ^2^ (207) = 516.39, *p* < .001, CFI = .969, RMSEA = .052 [90% CI = .045 - .059], SRMR = .025), which explains 58.4 percent of the variance (S8 Table).

The PAF reveals a conceptually meaningful three-factor structure with high internal consistencies for all three factors (Cronbach’s *α* > .80) and the aggregated 24-item scale (Cronbach’s *α* = .95). The first factor is labeled *ordinariness* (ORD) and comprises ten items (λ ≥ .51 and ≤ .81). The items describe perceptions of politicians as ordinary (e.g., “The politician is down-to-earth”), not aloof, and likely the people that one knows or could see walking down the street. The factor also comprises items with perceptions of candidates speaking honestly about their past and giving others a chance to understand their weaknesses and true selves. The second factor is termed *consistency* (CON) and consists of nine items (λ ≥ .45 and ≤ .74). The items loadings on this factor describe perceptions of politicians being true to themselves in all situations (e.g., “The politician consistently presents his/her true beliefs”) and holding on to their positions and values even when they face negative consequences. The factor also contains items on perceptions that politicians say what they think and do not mince matters. Five items (λ ≥ .45 and ≤ .71) constitute the third factor, which we label as *immediacy* (IMM). This factor consists of items on politicians’ spontaneity and emotionality and their openness about their lives, thoughts, and opinions. Finally, as expected, the three factors are not independent of each other but substantially correlated: *r* = .67 (ORD and CON), *r* = .53 (ORD and IMM), and *r* = .48 (CON and IMM).

#### Discussion

Survey findings from a German quota sample gave first insights in the factorial structure of the scale. They suggest that judgments of politicians’ authenticity are reflected in impressions from their ordinariness, consistency, and immediacy. Although each of the factors can be meaningfully interpreted as one out of the four concept dimensions in the literature, not all items loaded on their designated factor. Consequently, the factor structure of the item pool partly differs from the proposed structure in theory. While we expected the scale to consist of four separate dimensions, the analysis suggests a three-dimensional structure. Other than in the theoretical considerations, there are no separate intimacy and immediacy dimensions. Instead, some intimacy and immediacy items constitute the third factor, whereas others load on the ordinariness and consistency factors. We consider methodological and theoretical explanations for this.

A methodological explanation may be that the intimacy and immediacy items were not adequately formulated to measure the designated aspects. Since the items’ wording has been very close to the proposed definitions in the literature and was considered valid by the expert panel, we think that theoretical reasons apply here. The dimensions were proposed to conceptually distinguish between similar but different facets of political authenticity across different research perspectives [4]. Still, the distinctions of intimacy (direct glimpses into politicians’ private persona) and immediacy (undisguised presentations of the self) seem to be less salient in recipients’ actual perception processes. Our results imply that these theoretical distinctions do not comply with the often fast and frugal impression formation processes by recipients. When politicians share private thoughts, opinions, and feelings, citizens associate this together with spontaneous and emotional appearances as signs of politicians’ immediacy.

### Study III: Confirmatory factor analysis

Results from Study 2 suggested three factors for the 24 P-PA items. We included these items in a second online survey to test the scale in an independent sample and investigate its convergent and discriminant validity. We also calculated a nomological network to examine the relationship of the second-order factor authenticity with relevant concepts outlined in political communication literature.

#### Sample and procedures

We used a similar quota procedure as in the first survey (S5 Table). Respondents were again recruited by the *respondi AG* and received a financial incentive. Informed consent was obtained with the same procedure used in Study 1. After applying the same data processing steps as in the first sample (S4 Table), 1,210 cases remained for the analyses (age: *M* = 47.1, *SD* = 15.8, range = 18–74; 51.0% female). We randomized whether respondents answered the 24 authenticity items either regarding Olaf Scholz, a candidate from the Social Democratic Party of Germany (SPD), or Armin Laschet, a candidate from the Christian Democratic Union of Germany (CDU). The CDU is a center-right party, whereas the SPD is considered a center-left party. Both parties formed the governing coalition in Germany when the survey was conducted. Scholz and Laschet were each nominated by their parties to run for chancellor in the German federal election in 2021. The survey was conducted four months before the election. Respondents answered the political authenticity items for a candidate regardless of how well they knew the politician. They also answered on a four-point scale whether they knew the candidates and were excluded from the analysis when they did not know the candidate they evaluated (< 2). Together with the scale items, we measured the single item (“The politician is authentic”) and included measures for respondents’ party identification, their political interest, and their intention to vote for the candidate and their parties (see S7 Table for all measures). The questionnaire was pretested with a convenience sample (*N* = 16).

#### Data analysis

Item analyses showed a satisfactory level of item difficulty for all 24 items (*index p* ≥ .43 and ≤ .61). Several items were moderately skewed (< |.63|) or had moderate negative kurtosis (< 1). Mardia’s coefficients for multivariate skewness and kurtosis suggested that the data deviated from multivariate normality (skewness: *z* = 7667.15, *p* < 0.01; kurtosis: *z* = 95.30, *p* < 0.01). Due to the violations of the normality assumption, we calculated confirmatory factor analyses (CFA) with robust maximum likelihood estimation and the Satorra-Bentler scaled chi-square statistics using the R package *lavaan* [52]. The three factors ordinariness, consistency, and immediacy were modeled as first-order-latent constructs. The items were constrained to load only on the one factor they were assigned to in the PAF. The factors were allowed to correlate. The fit between the model and the data was evaluated using the chi-square test and fit indices with cut-off values recommended by Hu and Bentler [53]: An appropriate model fit was expected for comparative fit index (CFI) value close to .95, the root mean square error of approximation (RMSEA) lower than .06, and standardized root mean square residuals (SRMR) lower than .08.

#### Results

*First-order models*. We estimated a first-order CFA model with all 24 items, which exhibits fair fit to the data (rχ^2^ (249) = 1,338.82, *p* < .001, rCFI = .924, rRMSEA = .07 [90% CI = .066 - .073], SRMR = .045). All items (except imm_5) show high factor loadings (λ > .55) for the designated factor. Modification indices indicate potential cross-loadings for several items, suggesting that the model would improve if these items would not be constrained to load only on one factor. This raises questions about the dimensions’ discriminant validity due to items that were not assigned to their designated dimension in the EFA. Thus, we decided to exclude the one item with low factor loadings (imm_5) and the four items that showed the discrimination problems (con_11, imm_5, imm_1, imm_8).

A second CFA with the remaining 19 items was run. The model exhibits good fit between the model and the data (rχ^2^ (149) = 689.20, *p* < .001, rCFI = .950, rRMSEA = .06 [90% CI = .058 - .067], SRMR = .038). The number of items per dimension in this model differed between the three dimensions, ranging from 8 items (ordinariness) to 4 items (immediacy) per dimension. To provide a more balanced and parsimonious measure, we decided to remove further items from the ordinariness and consistency dimensions and to aim for a number of four items per factor in the final scale [54]. The items retained for the final scale were selected due to their theoretical relevance for the concept. Hence, we selected items reflecting central but different aspects of each dimension and excluded items with redundant content (ord_1, ord_2, con_3, con_6, con_9, int_3, imm_7). The third CFA model for the 12 items exhibits a good and improved model fit (rχ^2^ (51) = 210.66, *p* < .001, rCFI = .975, rRMSEA = .06 [90% CI = .050 - .066], SRMR = .033). All items loaded significantly and strongly on their designated dimension and the three factors were highly correlated (*r*_*ORD-CON*_ = .86, *r*_*CON-IMM*_ = .70, *r*_*ORD-IMM*_ = .76).

We calculated separate CFA models for the 12 items to test whether the three factor-model was more appropriate than one- or two-factor solutions. Given the high latent correlation between ordinariness and consistency, a two-factor model was tested in which the ordinariness and consistency items were set to load on one single latent factor. However, the two-factor model demonstrated worse fit compared with the three-factor model (rχ^2^ (53) = 431.17, *p* < .001, rCFI = .940, rRMSEA = .09 [90% CI = .080 - .096], SRMR = .044). As previous multi-item measures of political authenticity found items to load on a single latent factor, a single-factor model was also specified in which all items were set to load onto a global authenticity factor. This model fit worse to the data than the two- and three-factor solutions (rχ^2^ (54) = 866.13, *p* < .001, rCFI = .870, rRMSEA = .13 [90% CI = .121 - .136], SRMR = .066), supporting the assumption that the three-dimensional model is the best fit for the data.

**Fig 1:**
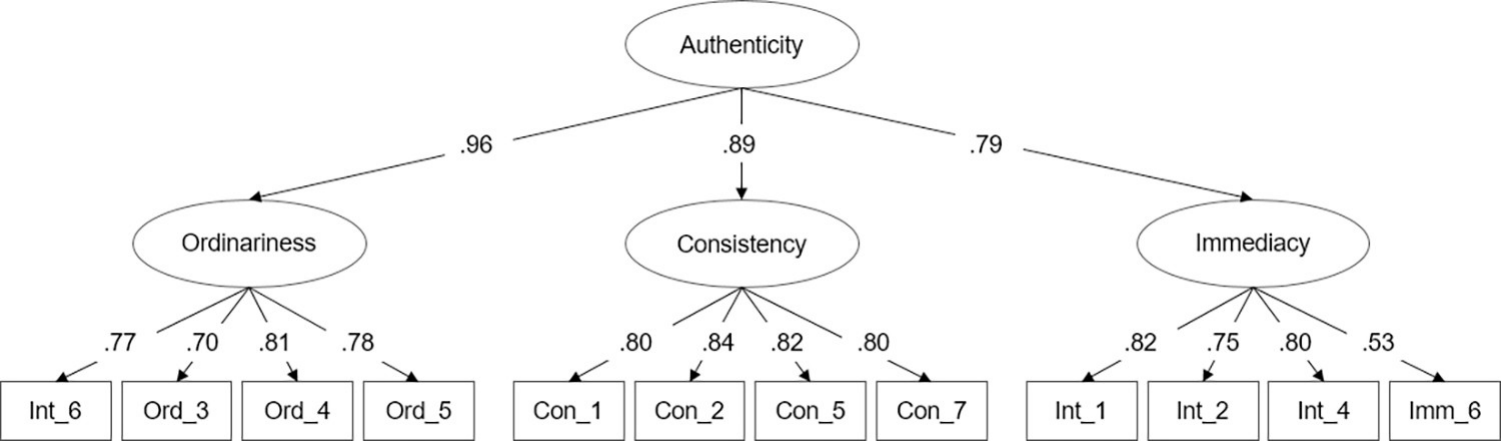
Confirmatory factor analysis of the perceived political authenticity scale. *Note*. Standardized path coefficients significant at *p* < .01. Based on data from *N* = 1,210 participants (Study III), rχ2 (51) = 210.66, *p* < .001, rCFI = .975, rRMSEA = .06 [90% CI = .050 - .066], SRMR = .033.

*Second-order model*. As outlined in theory, the factors are assumed to reflect a higher-order factor authenticity. Thus, a second-order model with authenticity as the higher-order construct was tested (Fig 1). The second-order factor allows analyzing the loadings for the higher-order construct and the construct’s relationship with relevant concepts outlined in political communication literature. Specifically, we investigate links of authenticity with plausible determinants and outcomes of authenticity, such as political attitudes and vote intentions. Before that, we calculated a model which only tested the relationship between the second-order factor and the single item (“The politician is authentic”). Results from the model (rχ^2^ (62) = 230.10, *p* < .001, rCFI = .977, rRMSEA = .054 [90% CI = .047 - .062], SRMR = .033) reveal a high correlation between the P-PA Scale and the single-item authenticity measure (*r* = .86, *p* < .001).

**Fig 2:**
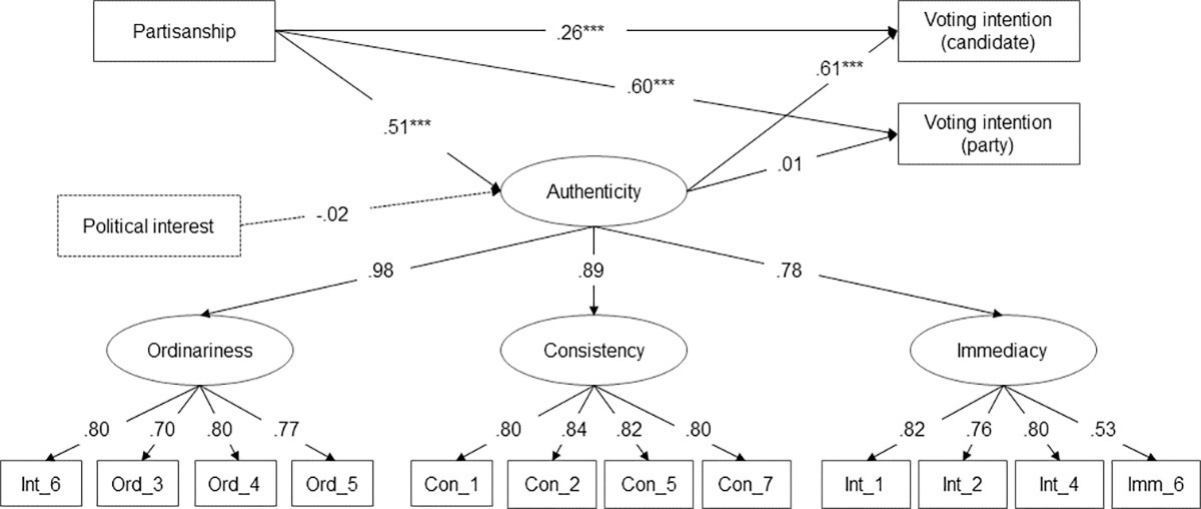
SEM assessing the construct validity of the perceived political authenticity scale. *Note*. Standardized path coefficients significant. Based on data from *N* = 1,210 participants (Study III), r*χ*^2^(97) = 303.830, *p* < .001, rCFI = .970, rRMSEA = .052, 90% CI = [.045, .059], and SRMR = .031. *** *p* < .001, **p < .01, *p < .05, ^#^*p* < .10.

*Nomological network*. The nomological network approach was applied to examine the overall theoretical context of the concept and “set forth the laws in which it occurs” ([55], p.290). Voting behavior was considered the overall theoretical context in which perceived political authenticity is embedded, suggesting relevant antecedents and outcomes of the concept. Therefore, perceived political authenticity was conceptualized as part of candidate evaluations, which are strongly influenced by partisanship and impact voting behavior [56]. Previous studies already identified party identification as a relevant predictor of perceived authenticity of specific politicians, showing that Democrats were more likely to perceive the Democratic candidate as authentic, whereas Republicans perceived the Republican candidate as more authentic [12,15]. Moreover, appearing authentic was shown to increase politicians’ chance of getting elected [14,15]. To test these theoretical assumptions, we computed a structural equation model (SEM) using *lavaan* [52]. Perceived political authenticity was regressed on partisanship in this model and set as a predictor for the intentions to vote for candidates and their parties (Fig 2). We also included political interest as a predictor in the model that was assumed to be unrelated to perceived political authenticity.

The nomological network demonstrates the expected positive relationships of the P-PA Scale with partisanship (*β* = .51, *p* < .001) and vote intention for the candidate (*β* = .61, *p* < .001). Perceived political authenticity is also shown to be unrelated to political interest (*β* = -.02, *p* = .59) and vote intention for a candidate’s party (*β* = .01, *p* = .84). These results suggest that the scale shows both convergent and discriminant validity. As suggested in theory, the scale is significantly correlated with partisanship and positively influences the intention to vote for a candidate (convergent validity). The results also demonstrate that perceived political authenticity of specific politicians is strongly related to party identification and candidate voting intention but still represents a distinct measure. The fact that authenticity—as expected—does not depend on political interest further supports this (discriminant validity).

#### Discussion

Results from a second and independent sample replicate the three dimensions of perceived political authenticity derived from the EFA in a restricted model. The number of items in the final scale was reduced to four items per dimension to balance the proportions of the three dimensions and to provide a parsimonious but still comprehensive 12-item measure that can easily be applied in different contexts (Table 1, see S10 Table for translated items). Regression analyses support the measure’s convergent and discriminant validity. Findings from the SEM with authenticity as a higher-order factor provide evidence for the scale’s relationship with relevant concepts and distinctiveness from other measures. Citizens perceive candidates as more authentic the more they identify with the candidate’s party and are more likely to vote for them when they appear more authentic. Still, perceived political authenticity did not predict the intention to vote for a candidate’s party. This lack of direct effects on party voting may be due to the inclusion of partisanship in the model, which has a plausible strong effect on the intention to vote for the party.

**Table 1:** Items of the final P-PA scale.

Dimension	Label *(old)*	Item (The politician …)	Reference
Ordinariness	ORD1 *(ord_4)*	is down-to-earth.	New item
	ORD2 *(ord_5)*	is not aloof.	New item
	ORD3 *(int_6)*	talks in a way that makes me feel familiar with him/her.	New item
	ORD4 *(ord_3)*	is likely the people you would see walking down the street.	Hall [43]
Consistency	CON1 *(con_1)*	presents positions consistent with his/her true beliefs.	Sweetser and Tedesco [38]
	CON2 *(con_2)*	consistently presents his/her true beliefs.	Sweetser and Tedesco [38]
	CON3 *(con_5)*	is true to him-/herself regardless of the situation.	Adapted from Becker [28]
	CON4 *(con_7)*	stands by his/her opinion even if it will cost him/her votes	Stiers et al. [15]
Immediacy	IMM1 *(int_1)*	speaks openly and honestly about his/her life.	Adapted from Becker [28]; Ilicic and Webster [42]
	IMM2 *(int_4)*	shares private thoughts, opinions, and feelings.	New item
	IMM3 *(int_2)*	allows others to participate in his/her private life.	New item
	IMM4 *(imm_6)*	often acts emotionally.	New item

*Note*. Items were measured on a 5-point Likert scale ranging from 1 (“I completely disagree”) to 5 (“I completely agree”).

A closer look at the perceived authenticity of the two candidates in the full sample shows almost identical mean values for the center-left candidate Olaf Scholz and the center-right candidate Armin Laschet but different values for the individual dimensions (S9 Table). Thus, Laschet was perceived as more immediate among the respondents, whereas Scholz appeared slightly more ordinary and consistent than his opponent. Moreover, bivariate correlations of a P-PA mean index with party identification, vote intention for the candidate, and vote intention for the party are stronger for Olaf Scholz (S9 Table). Thus, the association between perceived authenticity and party identification is stronger among Social Democrats (center-left) than Christian Democrats (center-right). As these examples demonstrate the scale’s general applicability and its potential to analyze interpersonal differences in authenticity perceptions and effects, the results also demand further research to elaborate these questions in more detail.

Finally, the third study reveals a strong correlation between the P-PA Scale and the single-item authenticity measure, indicating convergent validity of the new 12-item scale. We interpret this as further evidence that the scale does indeed measure the designated concept. The relationship of the single item with mean values for each of the P-PA Scale’s subdimensions (*r*_*ORD*_ = .79, *r*_*CON*_ = .76, *r*_*IMM*_ = .57) shows that the degree of variance in authenticity perceptions captured by the single item differs across the scale dimensions (S9 Table). Thus, respondents’ associations with the term “authentic” were more strongly correlated with aspects assigned to the ordinariness and consistency dimension. Using the single item has the consequence that the theoretically meaningful immediacy dimension of authenticity is less covered. Still, correlations between the single item and the analyzed attitudes and candidate evaluations in this study are close to those of the P-PA Scale, even though the single item’s correlations are somewhat lower in each case. We draw from this that the single item (“The politician is authentic”) shares large parts of the variance with the P-PA Scale. Thus, it may be used as an alternative instrument to measure perceived political authenticity when space is limited and when the examination of politicians’ authenticity and especially of different concept facets is not the primary research goal [57].

## General discussion

Authenticity is viewed as a crucial concept in political communication (e.g., Jones [3]). However, research on political authenticity had not yet agreed on a common definition of the concept and applied different instruments to measure perceptions of politicians’ authenticity, which have various limitations. Therefore, we define perceived political authenticity as a subjective assessment by citizens whether politicians appear as true to themselves and propose a new 12-item scale based on a conceptualization of political authenticity in communication literature. This article presents findings on the composition, performance, and validity of the scale and finds strong support for a three-dimensional factor structure.

The results of the three studies reveal the P-PA Scale as a robust and valid measure for authenticity perceptions. The items’ content validity was assessed with experts’ qualitative comments and with the quantitative content validity index. To our knowledge, this approach has not been applied in political communication research before and hence presents a novel approach for the quantification of content validity assessment in the discipline. Results of exploratory and constraint factor analyses suggest that perceived political authenticity is reflected in impressions from politicians’ ordinariness, consistency, and immediacy. This factor structure has been tested in two independent quota samples and complies with the proposed conception of political authenticity. Items from all four theoretical dimensions remained in the final scale. However, the scale structure deviates from the theory because intimacy and immediacy were not identified as separate empirical dimensions. Four intimacy and immediacy items together constitute a common factor, and three of these items were originally designed to represent a separate intimacy factor. Still, we decided to label this factor as immediacy because the four items describe impressions of politicians being authentic due to their openness about their lives and emotions. Whether intimacy and immediacy represent distinct empirical dimensions of performed and mediated political authenticity [4] remains an open question for future research on the concept. In case empirical testing of the dimensions in other national samples and studies analyzing candidates’ self-presentation or the news media’s construction of authenticity also reveal deviations from Luebke’s [4] conceptualization, the proposed structure should be reconsidered.

The P-PA Scale can advance research on political authenticity in several ways. First, it provides scholars with a valid instrument to analyze individual and group differences in authenticity perceptions in more detail. While single items measure subjective associations with the term “authentic”, the P-PA Scale consists of items derived from the theory that capture meaningful impressions of candidates’ authenticity. It allows not only to investigate whether citizens differ in their overall authenticity judgments but also to understand how these differences are formed and expressed in three dimensions of authenticity. Considering political authenticity as a driver for political success [13], the analysis of individual concept dimensions allows scholars to better understand the popularity of contemporary political leaders and why voters prefer certain candidates over others. Scholars can also apply the scale to investigate links between authenticity and populism. Previous research has shown that candidates from populist parties are perceived as more authentic [7]. Whether candidates from populist parties or those who use populist rhetoric style elements are more likely to score higher on specific authenticity dimensions would be an interesting question in understanding the success of populist politicians. Current notions of populism as an ideology that combines facets such as anti-elitism, people centrism, and restoring the people’s sovereignty suggest a plausible link with the ordinariness dimension of P-PA [58]. In addition, definitions of populism as a communication style with aspects such as intimacy and emotionality indicate a relationship to the intimacy dimension of authenticity [58].

The present research also has implications for the practice of political communication. Measuring politicians’ authenticity with the multidimensional P-PA Scale provides politicians and campaign managers with more information on how perceived authenticity among voters can be improved (e.g., during election campaigns). Candidates scoring low in one of the authenticity dimensions may increase their perceived authenticity and overall image among voters by strategically improving impressions for that facet. Future research should investigate potential differences in the construct dimensions between politicians from different parties. The two candidates from center-right and center-left parties evaluated in our second sample were perceived as equally authentic overall. However, Scholz was perceived as slightly more ordinary and consistent, whereas Laschet scored higher in immediacy. The causes for these variances may be numerous [12] and should be addressed in future studies.

Second, the instrument allows scholars and practitioners to better analyze the causes and consequences of perceived political authenticity. The scale and its dimensions correlate to varying degrees with other attitudinal and behavioral constructs, such as party identification and vote intention. Citizens’ evaluation of authenticity may also depend on the extent to which they consider the authenticity of politicians to be a relevant quality. Future research could test the influence of such a relative preference for authenticity over other candidate image variables such as competence or honesty on perceived political authenticity ratings. Still, one can assume that “authenticity is not entirely in the eye of the beholder” ([9], pp.866-867). For this reason, the scale can be used to identify further antecedents of perceived political authenticity, such as exposure to candidates’ performed authenticity. As social media is considered “a venue where politicians reveal their true selves” ([59], pp. 1–2), research on the effects of exposure to politicians’ performed authenticity in social media on the three dimensions is of interest [30]. Moreover, the scale is useful for understanding the effects of perceived authenticity on citizens’ candidate evaluations and political actions. Our studies show that it highly correlates with partisanship and vote intention. Future research should also test the relationship between perceived political authenticity and related measures for candidates’ integrity, sincerity, or trustworthiness. Research on the relative effects of authenticity compared to other image variables like competence and leadership qualities [18] could contribute to a better understanding of its relevance for candidate evaluations.

Third, the instrument relies on a new conceptualization of authenticity in politics and has only been validated for this purpose. We therefore assume that the scale is more appropriate for measuring politicians’ authenticity than existing scales for perceived authenticity of persons such as scientists [60], CEOs [61], and celebrities [42]. Still, if adapted to the particular context, we assume that the scale can be fruitful for analyzing perceived authenticity in other fields. As authenticity is considered a relevant trait in different social contexts [62], research should examine whether the instrument can be applied to analyze the perceived authenticity of other persons, such as journalists or social media influencers.

Besides these contributions, different limitations of the studies should be mentioned. First, our results rely on data from two online surveys of heterogeneous quota samples in Germany. Therefore, our findings are limited to this population. Although we expect the identified factor structure to apply across countries, specifics of the German political system and its politicians may have influenced the results [63]. Thus, the factor structure should be tested in other national contexts to be applicable for comparative research. Examining the latent correlations between the factors and authenticity in different national samples would test whether the dimensions have the same relevance for the higher-order construct across populations. For this purpose, the English scale version should be examined. Although the items were developed in English, and some were derived from prior international studies, only the German version of the items was tested (S10 Table). Second, our analyses focused on popular federal politicians in Germany at the time of the study. Respondents rated politicians only when they knew the politicians. Thus, it remains unclear whether the composition of the scale also applies in the same way for state politicians or less popular candidates. Finally, the main objective of this research was to develop and validate a new measurement instrument. The analyses relied on cross-sectional data to assess the construct’s validity. Therefore, this research does not confirm causal relationships between authenticity and related constructs. The direction of effects between authenticity and other constructs examined here (e.g., party identification) remains unclear and needs to be further explained by theory.

Despite these constraints, this article proposes a valuable new instrument to analyze and understand citizens’ perceptions of politicians’ authenticity. This research responds to observations that the outcome of elections increasingly depends on the images of political candidates among voters [64] and that being perceived as authentic plays an increasingly important role in this context [1]. It provides scholars and practitioners with a valuable tool to understand and explain the success (or failure) of political candidates. We hope that future research will further elaborate and apply the P-PA Scale and its dimensions to establish an instrument that provides valuable insights into the characteristics, determinants, and effects of perceived political authenticity.

## Supporting information

S1 Fig Sample 1: Screeplot for parallel analysis with 28 P-PA items. (TIF)

S2 Fig Sample 1: Screeplot for parallel analysis with 24 P-PA items. (TIF)

S1 Table Item pool after revisions. (DOCX)

S2 Table Content validity: Design and questionnaire (translated to English). (DOCX)

S3 Table Ratings of content validity in two rounds: Relevance for designated dimensions. (DOCX)

S4 Table Sample information. (DOCX)

S5 Table Sample distributions of sociodemographic variables (N after data preparation). (DOCX)

S6 Table Sample 1: Names, party affiliation, and popularity of evaluated politicians. (DOCX)

S7 Table List of item wordings in sample 1 and sample 2. (DOCX)

S8 Table Exploratory factor analyses (EFA) results. (DOCX)

S9 Table Pearson bivariate correlations between the P-PA scale mean and other constructs. (DOCX)

S10 Table Items of the final P-PA Scale (English and German version). (DOCX)

S1 Dataset Study II: EFA sample. (CSV)

S2 Dataset Study III: CFA sample. (CSV)
