# Supplementary figures and images for: Perceiving politicians as true to themselves: Development and validation of the perceived political authenticity scale
Source: PLoS One. 2023 May 24;18(5):e0285344. doi: 10.1371/journal.pone.0285344 (PMC10208464; doi:10.1371/journal.pone.0285344)

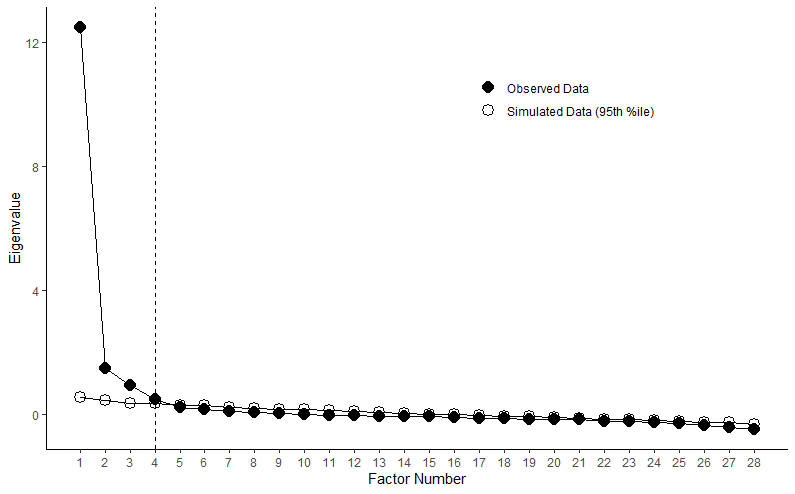

Supplement: S1 Fig — (TIF) [file pone.0285344.s001.tif]

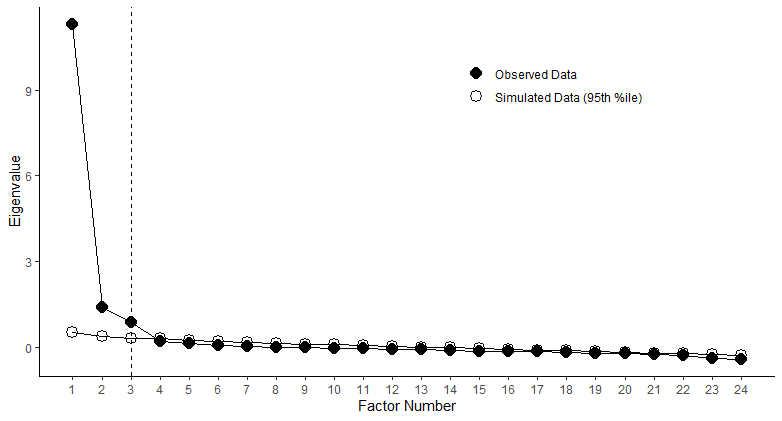

Supplement: S2 Fig — (TIF) [file pone.0285344.s002.tif]
