# Supplementary material for: Perceiving politicians as true to themselves: Development and validation of the perceived political authenticity scale
Source: PLoS One. 2023 May 24;18(5):e0285344. doi: 10.1371/journal.pone.0285344 (PMC10208464; doi:10.1371/journal.pone.0285344)
Supplement: S1 Table — (DOCX) [file pone.0285344.s003.docx]

# **S1 Table. Item pool after revisions**

| Label^1^ | Items [The politician …] | German Translation  [Der Politiker/die Politikerin …] | Reference |
| --- | --- | --- | --- |
| Con_1 | presents positions consistent with his/her true beliefs. | vertritt Positionen, die mit seinen/ihren wahren Ansichten übereinstimmen. | Sweetser and Tedesco (2014) |
| Con_2 | consistently presents his/her true beliefs. | stellt durchweg seine/ihre wahren Ansichten dar. | Sweetser and Tedesco (2014) |
| Con_3 | does what he/she says he/she will do. | handelt so, wie er/sie sagt, dass er/sie handeln wird. | Sweetser and Tedesco (2014) |
| Con_4_r | is a different person in public than in his/her private life. | ist in der Öffentlichkeit ein anderer Mensch als im Privaten. | Sweetser and Tedesco (2014) |
| Con_5 | is true to him-/herself regardless of the situation. *[original: People can count on Donald Trump being who he is regardless of the situation.]* | bleibt sich selbst unabhängig von der Situation treu. | Becker (2018) |
| Con_6 | acts consistent with his/her held values, even if others criticize or reject him/her for doing so. *[original: Tries to act in a manner that is consistent with his held values, even if others criticize or reject him for doing so.]* | handelt entsprechend seiner/ihrer Wertvorstellungen, selbst dann, wenn andere ihn/sie dafür kritisieren. | Becker (2018) |
| Con_7 | stands by his/her opinion even if it will cost him/her votes. *[original: won’t change his/her opinion just to get votes.]* | steht zu seiner Meinung selbst dann, wenn es ihn/sie Stimmen kostet. | Stiers et al. (2021) |
| Con_8 | behaves the same in public as in private. | verhält sich in der Öffentlichkeit genauso wie im privaten Bereich. | Stiers et al. (2021) |
| Con_9 | acts the way I expect him/her to act. | handelt so, wie ich es von ihm/ihr erwarte. | New item |
| Con_10 | acts the way I expect a politician to act. | handelt so, wie ich es von einem Politiker/einer Politikerin erwarte. | New item |
| Con_11 | keeps his/her word. | hält sein/ihr Wort. | New item |
| Con_12 | acts as I expect a person of his/her gender to act. | verhält sich so wie ich es von einer Person seines/ihres Geschlechts erwarte. | New item |
| Int_1 | speaks openly and honestly about his/her life. *[original: Cares about openness and honesty in close relationships with others.]* | berichtet offen über sein/ihr Leben. | Becker (2018); Ilicic and Webster (2016) |
| Int_2 | allows others to participate in his/her private life. | lässt andere an seinem/ihrem privaten Leben teilhaben. | New item |
| Int_3 | gives me a chance to understand his/her true self. | gibt mir die Möglichkeit, dass ich sein/ihr wahres Selbst verstehe. | New item |
| Int_4 | shares private thoughts, opinions, or feelings. | teilt private Gedanken, Ansichten oder Empfindungen. | New item |
| Int_5 | shows his real character to others. | offenbart anderen seinen/ihren wahren Charakter | New item |
| Int_6 | talks in a way that makes me feel familiar with him/her. | spricht in einer Art und Weise, die mir das Gefühl gibt, mit ihm/ihr persönlich vertraut zu sein. | New item |
| Int_7_r | does not reveal anything about his or her personal life. | gibt nichts aus seinem/ihren Privatleben preis. | New item |
| Int_8 | speaks sincerely about his past. | berichtet aufrichtig über seine/ihre Vergangenheit. | New item |

S1 Table. (continued)

| Ord_1 | gives me a chance to understand his/her weaknesses. *[original: wants people to understand his weaknesses.]* | gibt mir die Möglichkeit, seine/ihre Schwächen zu verstehen. | Ilicic and Webster (2016) |
| --- | --- | --- | --- |
| Ord_2 | is likely the people I personally know.  *[original: I personally know people who are like the people on the program.]* | ähnelt Menschen, die ich persönlich kenne. | Hall (2009) |
| Ord_3 | is likely the people you would see walking down the street. | ähnelt Menschen, die man auf der Straße trifft. | Hall (2009) |
| Ord_4 | is down-to-earth. | ist bodenständig. | New item |
| Ord_5 | is not aloof. | ist nicht abgehoben. | New item |
| Ord_6 | sometimes makes mistakes like everybody else. | macht manchmal Fehler, wie jeder andere auch. | New item |
| Ord_7 | is not always perfect. | ist nicht immer perfekt. | New item |
| Ord_8_r | appears flawless to me. | wirkt auf mich makellos. | New item |
| Ord_9 | appears fallible to me. | wirkt auf mich fehlbar. | New item |
| Ord_10_r | always thinks he/she is right. | glaubt immer im Recht zu sein. | New item |
| Imm_1 | has messages that reveal his/her true self. | hat Botschaften, die sein/ihr wahres Selbst offenbaren. | Sweetser and Tedesco (2014) |
| Imm_2 | appears genuine to me. | wirkt auf mich unverstellt. | New item |
| Imm_3 | places a good deal of importance on others understanding who he truly is. | legt Wert darauf, dass andere verstehen, wer er/sie wirklich ist. | Becker (2018) |
| Imm_4_r | is strongly influenced by the opinions of others. | lässt sich leicht durch die Meinung anderer beeinflussen. | Rosenblum et al. (2020) |
| Imm_5 | often acts spontaneously. | handelt oft spontan. | New item |
| Imm_6 | often acts emotionally. | handelt oft emotional. | New item |
| Imm_7 | does not mince matters. | nimmt kein Blatt vor den Mund. | New item |
| Imm_8 | says what he/she thinks. | sagt das, was er/sie denkt. | New item |
| Imm_9 | sometimes says things that are not considered politically correct. | sagt manchmal Dinge, die nicht unbedingt als politisch korrekt gelten. | New item |
| Imm_10_r | gives me the impression that he/she plans each of his/her steps in advance. | macht auf mich den Eindruck als plane er/sie jeden seiner/ihrer Schritte im Voraus. | New item |

*Note.* Items were measured on a 5-point Likert scale ranging from 1 (“I completely disagree) to 5 (I completely agree). ^1^ Con = designated dimension is consistency; Int = designated dimension is intimacy; ORD = designated dimension is ordinariness; Imm = designated dimension is immediacy. The small r indicates reverse coded items (e.g., item Con_4_r).
