# Supplementary material for: Perceiving politicians as true to themselves: Development and validation of the perceived political authenticity scale
Source: PLoS One. 2023 May 24;18(5):e0285344. doi: 10.1371/journal.pone.0285344 (PMC10208464; doi:10.1371/journal.pone.0285344)
Supplement: S2 Table — (DOCX) [file pone.0285344.s004.docx]

# **S2 Table. Content validity: Design and questionnaire (translated to English)**

We interviewed six expert judges (three female and three male) from four different universities in Germany and Switzerland in a first round.^[[1]](#footnote-1)^ The interviewees were professors and senior researchers in communication science or media psychology and had either expertise in empirical communication research (content experts) and/or experience in scale development and validation (method experts). The expert completed the following online questionnaire in which they gave qualitative feedback on items’ clarity, necessity, and comprehensiveness.

| Page 1:  Introduction  Overall definition | Dear participants,  thank you very much for participating in this survey. The survey is part of a study to develop and validate a scale to measure perceived political authenticity. An important step in scale development is to assess whether the developed items sufficiently and adequately represent the construct to be measured (content validity). For this project we have chosen you as an expert and ask you for your assessment of the relevance, completeness, and clarity of the individual items.  The study looks at the construct of perceived political authenticity. Perceived political authenticity describes a process in which individuals form subjective impressions of a politician in order to assess the extent to which the politician is true to him-/herself. Generally, politicians are perceived as more authentic when people have the impression that they reveal their true selves to others. For this purpose, we distinguish four dimensions of political authenticity: consistency, intimacy, ordinariness and immediacy. | |
| --- | --- | --- |
| Page 2:  Instructions | For each of the dimensions of authenticity, several items were developed to cover the different facets of the respective dimension and thus of perceived political authenticity. Each item is designed in such a way that it is only relevant for one of the four dimensions. On the following four pages you will find a list with the same 40 items. At the top of each page, however, you will find a different definition of authenticity in terms of one of the four dimensions (the same definition is shown again at the bottom of each page).  1) Please read the definition of perceived authenticity on each page first.  2) Please rate the extent to which an item is relevant to the understanding of authenticity shown above. Please use the rating scale (1 = not relevant to the definition; 2 = somewhat relevant to the definition; 3 = relevant to the definition; 4 = highly relevant to the definition).  3) If you have any comments about the completeness, relevance, or clarity of items, please note them at the bottom of each page. | |
| Page 3 (rotated):  Definition of authenticity as consistency | *To what extent do you consider the following items to be relevant for the definition of authenticity as consistency?*  Authenticity as consistency: Politicians are perceived as authentic when their actions appear as consistent **with their true views**. The impression of authenticity occurs when politicians **act in the same way** in different **situations or roles** and/or **over time**. Politicians are authentic when they act the way one **expects** him/her to act, given prior experience with him/her or with politicians in general. Politicians are less authentic when they **act inconsistently** or when their actions contradict my expectations.  The politician … | |
| Item order was rotated | Item 1 | not relevant \| somewhat relevant \| relevant \| highly relevant |
|  | Item 2 | not relevant \| somewhat relevant \| relevant \| highly relevant |
|  | … | ... |
|  | Item 40 | not relevant \| somewhat relevant \| relevant \| highly relevant |

S2 Table. (continued)

| Page 4 (rotated):  Definition of authenticity as intimacy | *To what extent do you consider the following items to be relevant for the definition of authenticity as intimacy?*  Authenticity as intimacy: Politicians are perceived as authentic when citizens have the impression that they know the true personality of politicians and their **private persona**. When politicians are **open and honest about their lives** and give **intimate insights** into their private lives to show others their true character, they are perceived authentic. Authentic politicians give others the feeling **that they are familiar with them**. Politicians are less authentic when they keep their private lives to themselves.  The politician … | |
| --- | --- | --- |
| Item order was rotated | Item 1 | not relevant \| somewhat relevant \| relevant \| highly relevant |
|  | Item 2 | not relevant \| somewhat relevant \| relevant \| highly relevant |
|  | … | ... |
|  | Item 40 | not relevant \| somewhat relevant \| relevant \| highly relevant |
| Page 5 (rotated):  Definition of authenticity as ordinariness | *To what extent do you consider the following items to be relevant for the definition of authenticity as ordinariness?*  Authenticity as ordinariness: Politicians are perceived as authentic when they appear **like ordinary people** like you and me. Perceived authenticity is associated with the impression that politicians are **down-to-earth** and close to the people. Authentic politicians are **fallible**, stand by their **weaknesses and flaws**, and are **not aloof**. Politicians are less authentic when they appear aloof or detached.  The politician … | |
| Item order was rotated | Item 1 | not relevant \| somewhat relevant \| relevant \| highly relevant |
|  | Item 2 | not relevant \| somewhat relevant \| relevant \| highly relevant |
|  | … | ... |
|  | Item 40 | not relevant \| somewhat relevant \| relevant \| highly relevant |
| Page 6 (rotated):  Definition of authenticity as immediacy | *To what extent do you consider the following items to be relevant for the definition of authenticity as immediacy?*  Authenticity as immediacy: Politicians are perceived as authentic when one has the impression that they **reveal their true self directly** and immediately to others. The impression of authenticity is created when politicians **do not pretend** or are influenced by others but say and do the things they actually believe. Authentic politicians act **spontaneously** and **emotionally** and do not mince words. Politicians are less authentic when their appearances seem strategically planned.  The politician … | |
| Item order was rotated | Item 1 | not relevant \| somewhat relevant \| relevant \| highly relevant |
|  | Item 2 | not relevant \| somewhat relevant \| relevant \| highly relevant |
|  | … | ... |
|  | Item 40 | not relevant \| somewhat relevant \| relevant \| highly relevant |
| Page 7:  Feedback on item clarity, necessity, and comprehen­siveness | Finally, we have three open questions for you about the relevance, clarity, and completeness of the scale items. For your orientation, you will find all four definitions of political authenticity listed below.  *From your point of view, are there any items that are difficult to understand and should be worded more clearly? If so, indicate the original item here and then write down your suggestions for improvement.*  *From your point of view, are there any items that should generally be excluded from the survey? If yes, please indicate the original item and the reason for its exclusion here.*  *From your point of view, are there items that would also be relevant for one of the definitions but are missing so far?* | |

1. Methodologist do not agree on the ideal number of experts for content validity assessment. Lynn (1986), for example, recommends to consult at least three experts, whereas Rubio et al. (2003) advise to interview 3 to 10 experts. [↑](#footnote-ref-1)
