# Supplementary material for: Perceiving politicians as true to themselves: Development and validation of the perceived political authenticity scale
Source: PLoS One. 2023 May 24;18(5):e0285344. doi: 10.1371/journal.pone.0285344 (PMC10208464; doi:10.1371/journal.pone.0285344)
Supplement: S3 Table — (DOCX) [file pone.0285344.s005.docx]

# **S3 Table. Ratings of content validity in two rounds: Relevance for designated dimensions**

| Ratings of six experts in round 1 (in brackets: ratings of three new experts on revised items in round 2) | | | | | | | | | |
| --- | --- | --- | --- | --- | --- | --- | --- | --- | --- |
| Label^1^ | Indicated as  relevant ≥ 3 | | Indicated as  not relevant ≤ 2 | | I-CVI/Ave | | Interpretation after round 1 | Interpretation after round 2 | S-CVI/Ave  after round 2 |
| **Con_1** | 4 |  | 2 |  | .67 |  | Appropriate | -- | Consistency  .92  (includes  9 Items) |
| **Con_2** | 4 |  | 2 |  | .67 |  | Appropriate | -- |  |
| **Con_3** | 6 |  | 0 |  | 1 |  | Appropriate | -- |  |
| **Con_4_r** | 3 | (3) | 3 | (0) | .50 | (1) | Needs revision | Appropriate |  |
| **Con_5** | 6 |  | 0 |  | 1 |  | Appropriate | -- |  |
| **Con_6** | 5 |  | 1 |  | .83 |  | Appropriate | -- |  |
| **Con_7** | 2 | (3) | 4 | (0) | .33 | (1) | Needs revision | Appropriate |  |
| Con_8 | 3 |  | 3 |  | .50 |  | Deleted | -- |  |
| **Con_9** | 3 | (3) | 3 | (0) | .50 | (1) | Needs revision | Appropriate |  |
| Con_10 | 1 |  | 5 |  | .17 |  | Deleted | -- |  |
| **Con_11** | 5 |  | 1 |  | .83 |  | Appropriate | -- |  |
| Con_12 | 1 |  | 5 |  | .17 |  | Deleted | -- |  |
| **Int_1** | 3 | (3) | 3 | (0) | .50 | (1) | Needs revision | Appropriate | Intimacy  .85  (includes 8 items) |
| **Int_2** | 5 |  | 1 |  | .83 |  | Appropriate | -- |  |
| **Int_3** | 5 |  | 1 |  | .83 |  | Appropriate | -- |  |
| **Int_4** | 6 |  | 0 |  | 1 |  | Appropriate | -- |  |
| **Int_5** | 4 |  | 2 |  | .67 |  | Appropriate | -- |  |
| **Int_6** | 5 |  | 1 |  | .83 |  | Appropriate | -- |  |
| **Int_7_r** | 6 |  | 0 |  | 1 |  | Appropriate | -- |  |
| **Int_8** | 4 |  | 2 |  | .67 |  | Appropriate | -- |  |
| **Ord_1** | 3 | (2) | 3 | (1) | .50 | (.67) | Needs revision | Appropriate | Ordinariness  .94  (includes 9 items) |
| **Ord_2** | 6 |  | 0 |  | 1 |  | Appropriate | -- |  |
| **Ord_3** | 6 |  | 0 |  | 1 |  | Appropriate | -- |  |
| **Ord_4** | 6 |  | 0 |  | 1 |  | Appropriate | -- |  |
| **Ord_5** | 6 |  | 0 |  | 1 |  | Appropriate | -- |  |
| **Ord_6** | 6 |  | 0 |  | 1 |  | Appropriate | -- |  |
| **Ord_7** | 5 |  | 1 |  | .83 |  | Appropriate | -- |  |
| **Ord_8_r** | 3 | (3) | 3 | (0) | .50 | (1) | Needs revision | Appropriate |  |
| **Ord_9** | 3 | (3) | 3 | (0) | .50 | (1) | Needs revision | Appropriate |  |
| Ord_10_r | 2 |  | 4 |  | .33 |  | Deleted | -- |  |
| **Imm_1** | 4 |  | 2 |  | .67 |  | Appropriate | -- | Immediacy  .86  (includes  7 items) |
| **Imm_2** | 5 |  | 1 |  | .83 |  | Appropriate | -- |  |
| Imm_3 | 1 |  | 5 |  | .17 |  | Deleted | -- |  |
| Imm_4_r | 3 | (1) | 3 | (2) | .50 | (.33) | Needs revision | Deleted |  |
| **Imm_5** | 6 |  | 0 |  | 1 |  | Appropriate | -- |  |
| **Imm_6** | 6 |  | 0 |  | 1 |  | Appropriate | -- |  |
| **Imm_7** | 6 |  | 0 |  | 1 |  | Appropriate | -- |  |
| **Imm_8** | 5 |  | 1 |  | .83 |  | Appropriate | -- |  |
| Imm_9 | 1 |  | 5 |  | .17 |  | Deleted | -- |  |
| **Imm_10_r** | 3 | (2) | 3 | (1) | .50 | (.67) | Needs revision | Appropriate |  |

*Note.* ^1^Items in bold were considered content valid. Con = designated dimension is consistency; Int = designated dimension is intimacy; ORD = designated dimension is ordinariness; Imm = designated dimension is immediacy. The small r indicates reverse coded items (e.g., item Con_4_r).
