# Supplementary material for: Perceiving politicians as true to themselves: Development and validation of the perceived political authenticity scale
Source: PLoS One. 2023 May 24;18(5):e0285344. doi: 10.1371/journal.pone.0285344 (PMC10208464; doi:10.1371/journal.pone.0285344)
Supplement: S4 Table — (DOCX) [file pone.0285344.s006.docx]

# **S4 Table. Sample Information**

The first survey was fielded from September 29 to October 6, 2020. The second survey was conducted between May 20 and June 02, 2021. We aimed at a sample size of 700 (sample 1) and of 1500 (sample 2) German adult participants, following a quota plan for age, gender, and education. Participants below the age of 18 and over the age of 74, respondents who were still in school, and who did not assign as male or female were screened out on the first survey page. We used quota stops for respondents if their demographics were already sufficiently covered. Interviews that were cancelled due to quota-stop or attention checks did not count into the targeted sample sizes.

| Sample size (N) | | Steps of data preparation |
| --- | --- | --- |
| Sample 1 | Sample 2 |  |
| 1,228 | 2,293 | Registered survey starts |
| 1,090 | 2,250 | Sample size after quota screen outs  (Age below 18 or above 74, Sex other than male or female, and still in school) |
| 700 | 1,510 | Sample size after automatized attention-check screen outs (Meade & Craig, 2012)  Two instructed response items (IRI) were used (Gummer et al., 2018). |
| 640 | 1,392 | Sample size after exclusion of “speeders”  (participants who needed less than half of the sample median time to complete the survey) (Greszki et al., 2015) |
| 559 | 1,267 | Sample size after listwise exclusion of all participants with missing values in one of the P-PA items and of people that did not know any politician at least well n_sample1_ = 81; people that did not know the politician at all n_sample2_ = 122) |
| 556 | 1,210 | Sample size after exclusion of “straight-liners”  (all participants who implausibly “straight-lined” the P-PA item-sets which included reverse-coded items; but not those participants who only checked the middle-category “3”, which was considered to be a plausible response pattern). The small number of identified straight-liners is because one attention check was among the P-PA items. Thus, several straight-liners were among the attention-check screen outs. |
| 556 | 1,210 | Final sample size used for analysis |
