# Supplementary material for: Perceiving politicians as true to themselves: Development and validation of the perceived political authenticity scale
Source: PLoS One. 2023 May 24;18(5):e0285344. doi: 10.1371/journal.pone.0285344 (PMC10208464; doi:10.1371/journal.pone.0285344)
Supplement: S5 Table — (DOCX) [file pone.0285344.s007.docx]

# **S5 Table. Sample distributions of sociodemographic variables (*N* after data preparation)**

|  | Absolute frequency | | Relative frequency in % | | Quotas: Population^1^ in % | | Discrepancy sample and population in % | | |
| --- | --- | --- | --- | --- | --- | --- | --- | --- | --- |
|  | Sample 1 | Sample 2 | Sample 1 | Sample 2 |  |  | Sample 1 | Sample 2 | |
| Age |  | |  | |  |  |  | | |
| 18-29 | 121 | 238 | 21.8 | 19.7 | 22.5 | | - 0.7 | | - 2.8 |
| 30-44 | 130 | 281 | 23.4 | 23.2 | 24.6 | | - 1.2 | | - 1.4 |
| 45-59 | 172 | 378 | 30.9 | 31.3 | 29.7 | | + 1.2 | | + 1.6 |
| 60-74 | 133 | 312 | 23.9 | 25.8 | 23.2 | | - 0.1 | | + 2.6 |
| Sex |  | |  | |  |  |  | | |
| female | 280 | 617 | 50.4 | 51.0 | 51.7 | | - 1.3 | | - 0.7 |
| male | 276 | 593 | 49.6 | 49.0 | 48.3 | | + 1.3 | | + 0.7 |
| Formal Education^2^ | | | | |  |  |  | | |
| low | 178 | 423 | 32.0 | 35.0 | 35.3 | | - 3.3 | | - 0.3 |
| medium | 171 | 379 | 30.8 | 31.3 | 30.7 | | + 0.1 | | + 0.6 |
| high | 207 | 408 | 37.2 | 33.7 | 34.0 | | + 3.2 | | - 0.3 |
| Total N | 556 | 1,210 | 100.0 | 100.0 | 100.0 | |  | | |

*Note.* ^1^ Population refers to the 2018 German adult population (Statistisches Bundesamt, 2021). ^2^ Low educated: finished school without certificate, lowest formal qualification of Germany’s secondary school system (“Hauptschule”); medium educated: intermediary, secondary qualification (“Realschule”); high educated: qualification, entitling holders to study (“Fachhochschulreife/Abitur”) (Greszki et al., 2015).
