# Supplementary material for: Perceiving politicians as true to themselves: Development and validation of the perceived political authenticity scale
Source: PLoS One. 2023 May 24;18(5):e0285344. doi: 10.1371/journal.pone.0285344 (PMC10208464; doi:10.1371/journal.pone.0285344)
Supplement: S6 Table — (DOCX) [file pone.0285344.s008.docx]

# **S6 Table. Sample 1: Names, party affiliation, and popularity of evaluated politicians**

| Politician^1^ (Party)^2^ | Government/ Opposition | Popularity^3^ in the sample | Frequency of  evaluation | |
| --- | --- | --- | --- | --- |
|  |  | (n = 640) | (n = 556) | |
|  |  | Mean (SD) | Frequency \| Share | |
| Angela Merkel (CDU/CSU) | Government | 3.25 (0.76) | 130 | 23.4% |
| Markus Söder (CDU/CSU) | Government | 2.81 (0.92) | 65 | 11.7% |
| Horst Seehofer (CDU/CSU) | Government | 2.84 (0.83) | 71 | 12.8% |
| Heiko Maas (SPD) | Government | 2.46 (0.89) | 35 | 6.3% |
| Franziska Giffey (SPD) | Government | 1.93 (0.90) | 13 | 2.4% |
| Olaf Scholz (SPD) | Government | 2.54 (0.92) | 45 | 8.1% |
| Alexander Gauland (AfD) | Opposition | 2.28 (0.94) | 28 | 5.0% |
| Alice Weidel (AfD) | Opposition | 2.04 (0.98) | 21 | 3.8% |
| Gregor Gysi (Die Linke) | Opposition | 2.54 (0.93) | 55 | 9.9% |
| Robert Habeck (Bündnis 90/Die Grünen) | Opposition | 2.12 (1.01) | 22 | 4.0% |
| Claudia Roth (Bündnis 90/Die Grünen) | Opposition | 2.44 (0.92) | 38 | 6.8% |
| Christian Lindner (FDP) | Opposition | 2.50 (0.93) | 33 | 6.0% |
| Total |  | 2.48 (0.37) |  | - |

*Note.* ^1^All politicians were among the best-known German federal politicians at the time of the study (infratest dimap, 2020); ^2^ CDU/CSU: Christian Democratic Union/Christian Social Union, SPD: Social Democratic Party of Germany, AfD: Alternative for Germany, Die Linke: The Left, Bündnis 90/Die Grünen: Alliance ‘90/Greens Party. ^3^ Mean value of responses to the question: “Please indicate how well you know the following politicians from the media.” [1 = not at all; 2 = somewhat; 3 = well; 4 = very well].
