# Supplementary material for: Perceiving politicians as true to themselves: Development and validation of the perceived political authenticity scale
Source: PLoS One. 2023 May 24;18(5):e0285344. doi: 10.1371/journal.pone.0285344 (PMC10208464; doi:10.1371/journal.pone.0285344)
Supplement: S7 Table — (DOCX) [file pone.0285344.s009.docx]

# **S7 Table. List of item wordings in sample 1 and sample 2**

| **Items/Scale** | **Item wording *[English translation]*** | **Response codes** | **Sample** |
| --- | --- | --- | --- |
| Popularity of politicians | Bitte geben Sie an, wie gut Sie die folgenden Politikerinnen und Politiker jeweils aus den Medien kennen.  (Angela Merkel (CDU/CSU), Markus Söder (CDU/CSU), Horst Seehofer (CDU/CSU), Heiko Maas (SPD), Franziska Giffey (SPD), Olaf Scholz (SPD), Alexander Gauland (AfD), Alice Weidel (AfD), Gregor Gysi (Die Linke), Robert Habeck (Bündnis 90/Die Grünen), Claudia Roth (Bündnis 90/Die Grünen), Christian Lindner (FDP))  *[Please indicate how well you know the following politicians from the media.]* | 1 (überhaupt nicht), 2 (ein wenig), 3 (gut), 4 (sehr gut)  *1 (not at all), 2 (somewhat), 3 (well), 4 (very well)* | Sample 1 |
|  | Wie gut, würden Sie sagen, kennen Sie den bewerteten Politiker aus den Medien? Ich kenne den Politiker …  (Armin Laschet (CDU)/Olaf Scholz (SPD))  *[How well would you say you know the rated politician from the media? I know the politician …]* | 1 (überhaupt nicht), 2 (ein wenig), 3 (gut), 4 (sehr gut)  *1 (not at all), 2 (somewhat), 3 (well),  4 (very well)* | Sample 2 |
| Party identification  (Halmburger et al., 2019) | In Deutschland fühlen sich viele Menschen einer oder mehreren politischen Parteien verbunden, selbst wenn sie auch ab und zu eine andere Partei wählen. Wie ist das bei Ihnen: Wie sehr fühlen Sie sich den nachfolgenden Parteien verbunden?  (CDU/CSU, SPD, AfD, Bündnis 90/Die Grünen, FDP, Die Linke)  *[Most people feel connected to a political party in Germany even if they decide to vote for a different party from time to time. What about you: How much do you feel connected to the following parties?]* | 1 (sehr schwach) - 6 (sehr stark)  *1 (not at all) - 6 (very strong)* | Sample 2 |
| Political interest (Mader et al., 2020) | Wie stark interessieren Sie sich im Allgemeinen für Politik?  *[Quite generally, how interested are you in politics?]* | 1 (überhaupt nicht) - 5 (sehr stark)  *1 (not at all) - 5 (very interested)* | Sample 2 |

S7 Table. (continued)

| **Items/Scale** | **Item wording *[English translation]*** | **Response codes** | **Sample** |
| --- | --- | --- | --- |
| Vote intention  candidate | Mal angenommen, Sie könnten die eben von Ihnen bewertete Person direkt in ein politisches Amt wählen (z. B. Bundeskanzler). Wie wahrscheinlich wäre es, dass Sie diese Person wählen?  *[Assume you could directly elect the person you just evaluated to political office (e.g., Chancellor). How likely would it be that you would vote for this person?]* | 1 (sehr unwahrscheinlich) -  11 (sehr wahrscheinlich)  *1 (very unlikely) - 11 (very likely)* | Sample 2 |
| Vote intention party (Mader et al., 2020) | Wenn am nächsten Sonntag Bundestagswahl wäre, welche Partei würden Sie dann wählen?  (CDU/CSU; SPD; AfD; FDP; Die Linke; Bündnis 90/Die Grünen; andere Partei, und zwar: __; weiß nicht; Ich würde nicht wählen gehen)  *[If there were a federal election next Sunday, which party would you elect with your second vote?]* |  | Sample 2 |
| Authenticity single item (Hahl et al., 2018) | Der Politiker ist authentisch.  *[The politician is authentic.]* | 1 (ich stimme überhaupt nicht zu) -  5 (ich stimme voll und ganz zu)  *1 (I completely disagree) -  5 (I completely agree)* | Sample 2 |
| Gender | Bitte geben Sie an, welchem Geschlecht Sie sich zugehörig fühlen.  *[Please indicate the gender you feel you belong to.]* |  | Sample 1 &  Sample2 |
| Age | Tragen Sie hier bitte ihr Alter ein:  *[Please fill in your age here:]* |  |  |
| Education (Greszki et al., 2015) | Was ist Ihr höchster Schulabschluss? (Noch in schulischer Ausbildung; Ohne allgemeinen Schulabschluss; Haupt-/(Volks-)schulabschluss, Realschule oder gleichwertiger Abschluss, Fachhoschul- oder Hochschulreife)  *[What is you highest level of education?]* |  |  |

**References**

Greszki, R., Meyer, M., & Schoen, H. (2015). Exploring the effects of removing “too fast” responses and respondents from web surveys. *Public Opinion Quarterly*, *79*(2), 471–503.

Hahl, O., Kim, M., & Zuckerman Sivan, E. W. (2018). The authentic appeal of the lying demagogue: Proclaiming the deeper truth about political illegitimacy. *American Sociological Review*, *83*(1), 1–33.

Halmburger, A., Rothmund, T., Baumert, A., & Maier, J. (2019). Trust in politicians—Understanding and measuring the perceived trustworthiness of specific politicians and politicians in general as multidimensional constructs. In E. Bytzek, M. Steinbrecher, & U. Rosar (Eds.), *Wahrnehmung – Persönlichkeit – Einstellungen [Perception - Personality - Attitudes]* (pp. 235–302). Springer Fachmedien Wiesbaden.

Mader, M., Pesthy, M., & Schoen, H. (2020). Conceptions of national identity, turnout and party preference: Evidence from Germany. *Nations and Nationalism.* Advance online publication.
