# Supplementary material for: Perceiving politicians as true to themselves: Development and validation of the perceived political authenticity scale
Source: PLoS One. 2023 May 24;18(5):e0285344. doi: 10.1371/journal.pone.0285344 (PMC10208464; doi:10.1371/journal.pone.0285344)
Supplement: S8 Table — (DOCX) [file pone.0285344.s010.docx]

# **S8 Table. Exploratory factor analyses (EFA) results**

|  | 1 | 2 | 3 | *h*² |
| --- | --- | --- | --- | --- |
| **Ordinariness** (*α* = .927) |  |  |  |  |
| is down-to-earth. (Ord_4) | .81 |  |  | .68 |
| talks in a way that makes me feel familiar with him/her. (Int_6) | .74 |  |  | .66 |
| is not aloof. (Ord_5) | .71 |  |  | .62 |
| keeps his/her word. (Con_11) | .70 |  |  | .69 |
| speaks sincerely about his/her past. (Int_8) | .62 |  |  | .54 |
| is likely the people you would see walking down the street. (Ord_3) | .60 |  |  | .52 |
| gives me a chance to understand his/her weaknesses. (Ord_1) | .57 |  |  | .55 |
| is likely the people I personally know. (Ord_2) | .56 |  |  | .52 |
| acts the way I expect him/her to act. (Con_9) | .52 |  |  | .52 |
| gives me a chance to understand his/her true self. (Int_3) | .51 |  |  | .67 |
| **Consistency** (*α* = .931) |  |  |  |  |
| is true to him-/herself regardless of the situation. (Con_5) |  | .74 |  | .67 |
| acts consistent with his/her held values, even if others criticize or reject him/her for doing so. (Con_6) |  | .72 |  | .59 |
| does not mince matters. (Imm_7) |  | .72 |  | .59 |
| consistently presents his/her true beliefs. (Con_2) |  | .69 |  | .69 |
| stands by his/her opinion even if it will cost him/her votes. (Con_7) |  | .69 |  | .65 |
| presents positions consistent with his/her true beliefs. (Con_1) |  | .66 |  | .62 |
| says what he/she thinks. (Imm_8) |  | .60 |  | .62 |
| does what he/she says he/she will do. (Con_3) |  | .55 |  | .66 |
| has messages that reveal his/her true self. (Imm_1) |  | .45 |  | .44 |
| **Immediacy** (*α* = .804) |  |  |  |  |
| often acts spontaneously. (Imm_5) |  |  | .71 | .45 |
| allows others to participate in his/her private life. (Int_2) |  |  | .70 | .59 |
| speaks openly and honestly about his/her life. (Int_1) |  |  | .67 | .65 |
| often acts emotionally. (Imm_6) |  |  | .61 | .38 |
| shares private thoughts, opinions, and feelings. (Int_4) |  |  | .45 | .46 |
| **Eigenvalue** | 5.92 | 5.30 | 2.79 |  |
| **Explained variance** | 24.7% | 22.1% | 11.6% |  |
| **Cumulative variance** | 24.7% | 46.7% | 58.4% |  |

*Note. n* = 556; Principal axis factor analysis (PAF) with oblique (Promax) rotation; factor loadings λ < .40 have been omitted.
