# Supplementary material for: Perceiving politicians as true to themselves: Development and validation of the perceived political authenticity scale
Source: PLoS One. 2023 May 24;18(5):e0285344. doi: 10.1371/journal.pone.0285344 (PMC10208464; doi:10.1371/journal.pone.0285344)
Supplement: S9 Table — (DOCX) [file pone.0285344.s011.docx]

# **S9 Table. Pearson bivariate correlations between the P-PA scale mean and other constructs**

|  | *α* | *M* | *SD* | 1. | 2. | 3. | 4. | 5. | 6. | 7. | 8. |
| --- | --- | --- | --- | --- | --- | --- | --- | --- | --- | --- | --- |
| **1. Full P-PA Scale** | **.92** | **2.65** | **.82** | -- | **.79** | **.91** | **.90** | **.82** | **.47** | **.68** | **.29** |
| Armin Laschet (CDU) | .92 | 2.66 | .82 |  | .78 | .91 | .88 | .82 | .36 | .66 | .19 |
| Olaf Scholz (SPD) | .92 | 2.64 | .82 |  | .81 | .92 | .91 | .81 | .60 | .71 | .40 |
| **2. Single item** |  | 2.75 | 1.16 | **.79** | -- | **.76** | **.75** | **.57** | **.43** | **.64** | **.26** |
| Armin Laschet (CDU) |  | 2.70 | 1.15 | .78 |  | .74 | .71 | .60 | .31 | .60 | .18 |
| Olaf Scholz (SPD) |  | 2.80 | 1.18 | .81 |  | .79 | .79 | .55 | .56 | .68 | .36 |
| **3. Ordinariness** | **.85** | **2.75** | **.99** | **.91** | **.76** | -- | **.74** | **.63** | **.47** | **.63** | **.28** |
| Armin Laschet (CDU) | .85 | 2.70 | .99 | .91 | .74 |  | .70 | .68 | .35 | .59 | .18 |
| Olaf Scholz (SPD) | .85 | 2.79 | 1.00 | .92 | .79 |  | .79 | .61 | .60 | .67 | .40 |
| **4. Consistency** | **.89** | **2.82** | **.98** | **.90** | **.75** | **.74** | -- | **.60** | **.43** | **.63** | **.27** |
| Armin Laschet (CDU) | .88 | 2.80 | .98 | .88 | .71 | .70 |  | .61 | .31 | .59 | .17 |
| Olaf Scholz (SPD) | .89 | 2.85 | .97 | .91 | .79 | .79 |  | .60 | .56 | .67 | .39 |
| **5. Immediacy** | **.81** | **2.39** | **.82** | **.83** | **.57** | **.63** | **.60** | -- | **.34** | **.47** | **.20** |
| Armin Laschet (CDU) | .80 | 2.49 | .82 | .85 | .60 | .68 | .61 |  | .29 | .50 | .14 |
| Olaf Scholz (SPD) | .82 | 2.27 | .81 | .81 | .55 | .61 | .60 |  | .41 | .48 | .25 |
| **6. Party identification** |  | **2.92** | **1.58** | **.47** | **.43** | **.47** | **.43** | **.34** | -- | **.55** | **.60** |
| Armin Laschet (CDU) |  | 2.94 | 1.63 | .36 | .31 | .35 | .31 | .29 |  | .44 | .60 |
| Olaf Scholz (SPD) |  | 2.91 | 1.53 | .60 | .56 | .60 | .56 | .41 |  | .67 | .60 |
| **7. Vote intention (candidate)** |  | **4.09** | **3.16** | **.68** | **.64** | **.68** | **.63** | **.47** | **.55** | -- | **.42** |
| Armin Laschet (CDU) |  | 3.80 | 3.06 | .66 | .60 | .65 | .59 | .50 | .44 |  | .33 |
| Olaf Scholz (SPD) |  | 4.40 | 3.24 | .71 | .68 | .71 | .67 | .48 | .67 |  | .52 |
| **8. Vote intention (party)** |  | - | - | **.29** | **.26** | **.28** | **.27** | **.20** | **.60** | **.42** | -- |
| Armin Laschet (CDU) |  | **-** | **-** | .19 | .18 | .18 | .17 | .14 | .60 | .33 |  |
| Olaf Scholz (SPD) |  | **-** | **-** | .40 | .36 | .40 | .39 | .25 | .60 | .52 |  |

*Note.* All correlations are significant on the level *p* < .001.
