# Supplementary material for: Perceiving politicians as true to themselves: Development and validation of the perceived political authenticity scale
Source: PLoS One. 2023 May 24;18(5):e0285344. doi: 10.1371/journal.pone.0285344 (PMC10208464; doi:10.1371/journal.pone.0285344)
Supplement: S10 Table — (DOCX) [file pone.0285344.s012.docx]

# S10 Table. Items of the final P-PA Scale (English and German version)

| Dimension | Label  *(old)* | Item (The politician …) | German version  (Der Politiker/Die Politikerin …) | Reference |
| --- | --- | --- | --- | --- |
| Ordinariness | ORD1 *(Ord_4)* | is down-to-earth. | ist bodenständig. | New item |
|  | ORD2 *(Ord_5)* | is not aloof. | ist nicht abgehoben. | New item |
|  | ORD3  *(Int_6)* | talks in a way that makes me feel familiar with him/her. | spricht in einer Art und Weise, die mir das Gefühl gibt, mit ihm/ihr persönlich vertraut zu sein. | New item |
|  | ORD4 *(Ord_3)* | is likely the people you would see walking down the street. | ähnelt Menschen, die man auf der Straße trifft. | Hall (2009) |
| Consistency | CON1 *(Con_1)* | presents positions consistent with his/her true beliefs. | vertritt Positionen, die mit seinen/ihren wahren Ansichten übereinstimmen. | Sweetser and Tedesco (2014) |
|  | CON2 *(Con_2)* | consistently presents his/her true beliefs. | stellt durchweg seine/ihre wahren Ansichten dar. | Sweetser and Tedesco (2014) |
|  | CON3 *(Con_5)* | is true to him-/herself regardless of the situation. | bleibt sich selbst unabhängig von der Situation treu. | Adapted from Becker (2018) |
|  | CON4 *(Con_7)* | stands by his/her opinion even if it will cost him/her votes | steht zu seiner Meinung selbst dann, wenn es ihn/sie Stimmen kostet. | Stiers et al. (2021) |
| Immediacy | IMM1 *(Int_1)* | speaks openly and honestly about his/her life. | berichtet offen über sein/ihr Leben. | Adapted from Becker (2018); Ilicic and Webster (2016) |
|  | IMM2 *(Int_4)* | shares private thoughts, opinions, and feelings. | teilt private Gedanken, Ansichten oder Empfindungen. | New item |
|  | IMM3 *(Int_2)* | allows others to participate in his/her private life. | lässt andere an seinem/ihrem privaten Leben teilhaben. | New item |
|  | IMM4 *(Imm_6)* | often acts emotionally. | handelt oft emotional. | New item |

*Note.* Items were measured on a 5-point Likert scale ranging from 1 (“I completely disagree”) to 5 (“I completely agree”).

**References**

Becker, A. B. (2018). Live from New York, it’s Trump on Twitter! The effect of engaging with Saturday Night Live on perceptions of authenticity and the salience of trait ratings. *International Journal of Communication*, *12*, 1736–1757.

Greszki, R., Meyer, M., & Schoen, H. (2015). Exploring the effects of removing “too fast” responses and respondents from web surveys. *Public Opinion Quarterly*, *79*(2), 471–503.

Gummer, T., Roßmann, J., & Silber, H. (2018). Using instructed response items as attention checks in web surveys: Properties and implementation. *Sociological Methods & Research*, *50*(1), 238-264.

Hahl, O., Kim, M., & Zuckerman Sivan, E. W. (2018). The authentic appeal of the lying demagogue: Proclaiming the deeper truth about political illegitimacy. *American Sociological Review*, *83*(1), 1–33.

Hall, A. (2009). Perceptions of the authenticity of reality programs and their relationships to audience involvement, enjoyment, and perceived learning. *Journal of Broadcasting & Electronic Media*, *53*(4), 515–531.

Halmburger, A., Rothmund, T., Baumert, A., & Maier, J. (2019). Trust in politicians—Understanding and measuring the perceived trustworthiness of specific politicians and politicians in general as multidimensional constructs. In E. Bytzek, M. Steinbrecher, & U. Rosar (Eds.), *Wahrnehmung – Persönlichkeit – Einstellungen [Perception - Personality - Attitudes]* (pp. 235–302). Springer Fachmedien Wiesbaden.

Ilicic, J., & Webster, C. M. (2016). Being true to oneself: Investigating celebrity brand authenticity. *Psychology & Marketing*, *33*(6), 410–420.

infratest dimap (2020). ARD-DeutschlandTREND Februar 2020: Studie zur politischen Stimmung im Auftrag der ARD-Tagesthemen und der Tageszeitung DIE WELT [A study on the political opinion on behalf of the ARD tagesschau and the newspaper Die WELT].

Lynn, M. R. (1986). Determination and quantification of content validity. *Nursing Research*, *35*(6), 382–386.

Mader, M., Pesthy, M., & Schoen, H. (2020). Conceptions of national identity, turnout and party preference: Evidence from Germany. *Nations and Nationalism.* Advance online publication.

Meade, A. W., & Craig, S. B. (2012). Identifying careless responses in survey data. *Psychological Methods*, *17*(3), 437–455.

Rosenblum, M., Schroeder, J., & Gino, F. (2020). Tell it like it is: When politically incorrect language promotes authenticity. *Journal of Personality and Social Psychology*, *119*(1), 75–103.

Rubio, D. M., Berg-Weger, M., Tebb, S. S., Lee, E. S., & Rauch, S. (2003). Objectifying content validity: Conducting a content validity study in social work research. *Social Work Research*, *27*(2), 94–104.

Statistisches Bundesamt. (2021). *Deutschland, Jahre, Geschlecht, Altersgruppen, Allgemeine Schulausbildung*. https://www-genesis.destatis.de/genesis//online?operation=table&code=12211-0040&bypass=true&levelindex=0&levelid=1622887361073#abreadcrumb

Stiers, D., Larner, J., Kenny, J., Breitenstein, S., Vallée-Dubois, F., & Lewis-Beck, M. (2021). Candidate authenticity: ‘To thine own self be true’. *Political Behavior*, *43*, 1181–1204.

Sweetser, K. D., & Tedesco, J. C. (2014). Effects of bipartisanship messaging and candidate exposure on the political organization–public relationship. *American Behavioral Scientist*, *58*(6), 776–793.
